# Decline in Sensory Integration in Old Age and Its Related Functional Brain Connectivity Correlates Observed during a Virtual Reality Task

**DOI:** 10.3390/brainsci14080840

**Published:** 2024-08-21

**Authors:** Satoru Inagaki, Hirokazu Matsuura, Kazuki Sakurai, Ludovico Minati, Natsue Yoshimura

**Affiliations:** 1 Human Centered Science and Biomedical Engineering, Department of Computer Science, School of Computing, Tokyo Institute of Technology, Tokyo 226-8501, Japan; inagaki.s.af@m.titech.ac.jp; 2 Information and Communications Engineering, Department of Information and Communications Engineering, School of Engineering, Tokyo Institute of Technology, Tokyo 226-8501, Japan; matsuura@brain.c.titech.ac.jp (H.M.); sakurai@brain.c.titech.ac.jp (K.S.); 3 Institute of Innovative Research, Tokyo Institute of Technology, Tokyo 226-8501, Japan; lminati@ieee.org; 4 Center for Mind/Brain Science, University of Trento, 38122 Trento, Italy; 5 School of Life Science and Technology, University of Electronic Science and Technology of China, Chengdu 610056, China; 6 School of Computing, Tokyo Institute of Technology, Tokyo 226-8501, Japan

**Keywords:** sensory integration, aging, rsFC, reaching task, virtual reality

## Abstract

Sensory integration is an essential human function whose decline impacts quality of life, particularly in older adults. Herein, we propose an arm-reaching task based on a virtual reality head-mounted display system to assess sensory integration in daily life, and we examined whether reaching task performance was associated with resting-state functional connectivity (rsFC) between the brain regions involved in sensory integration. We hypothesized that declining sensory integration would affect performance during a reaching task with multiple cognitive loads. Using a task in which a young/middle-aged group showed only small individual differences, older adults showed large individual differences in the gap angle between the reaching hand and the target position, which was used to assess sensory integration function. Additionally, rsfMRI data were used to identify correlations between rsFC and performance in older adults, showing that performance was correlated with connectivity between the primary motor area and the left inferior temporal gyrus and temporo-occipital region. Connectivity between areas is related to visuomotor integration; thus, the results suggest the involvement of visuomotor integration in the decline of sensory integration function and the validity of the gap angle during this VR reaching task as an index of functional decline.

## 1. Introduction

Sensory integration is an important function supporting the quality of human life [1]. Even trivial daily movements, such as reaching for an object, are based on the integration of multiple sources of sensory information, including visual and proprioceptive information, and a decline in function affects the quality of life, especially in older adults [2]. In recent years, extensive studies have been conducted worldwide to elucidate the mechanisms underlying age-related decline [2,3].

Most studies have used performance [4,5], biological signals [6], and brain activity measurements [7,8,9] during motor tasks as indices to investigate the relationship between function and age. A representative example of a motor task is one that combines arm reaching (extending the arm from a fixed position toward a target) and a visuomotor rotation paradigm; using such a task, the ability to detect a gap (mismatch) between visual and proprioceptive feedback has been shown to decline with age [4,5]. In addition, muscle and brain activity signals measured during other motor tasks such as walking and tool manipulation show age-related decreases in reaction time and changes in activity in the visual, parietal, and motor cortical areas [6,7,8,9]. However, such indices are task-dependent, specifically requiring motor tasks [10]; therefore, the use of resting-state functional magnetic resonance imaging (rsfMRI) [5,11,12,13] to investigate the brain functional networks underlying sensory integration has attracted increasing interest in recent years. In particular, the functional connectivity between brain regions has been extensively studied, including the relationship between age-related decline in motor performance and resting-state functional connectivity (rsFC) across various age groups [11,13] and between motor performance and rsFC in children [12]. These results indicate that rsFC is a useful predictor of exercise performance.

Based on these prior results, in the present study, we analyzed performance during a motor task that combined arm-reaching and visuomotor rotation paradigms using the precise musculoskeletal robot KINARM as a sensory integration task, along with rsfMRI data. The results demonstrated differences in the rsFC between motor-related regions associated with the ability to detect visuomotor rotation between participants in their 20s and 60s [5]. These findings suggest that regular monitoring of performance in this motor task may allow the early detection of sensory integration decline in old age.

To achieve routine detection, it is necessary to develop a system using economical devices, such as commercially available personal computers and smartphones, rather than relying on large and expensive research equipment such as the KINARM. Among these, virtual reality (VR) systems, which have been increasingly adopted in recent years, are gaining increasing attention for their potential in the rehabilitation of patients with stroke and spinal cord injury [14,15]. Notably, VR systems allow the execution of motor tasks without the participants directly viewing their hands, making it possible to design sensorimotor integration tasks similar to those conducted using KINARM. However, the design of these motor tasks must consider the differences between the VR systems and KINARM. In the present study, based on prior research, we employed a visuomotor rotation task as the motor task, while adopting different performance metrics that considered the characteristics of VR head-mounted displays (VR-HMD), as well as the potential for home use. The gap-discrimination performance index used in conventional tasks is effective only for experiments performed by fixing the face and hand positions in a known manner with a precise exoskeleton robot, such as KINARM. Accordingly, considering that individuals freely move their head and hands when using a VR-HMD, we instead used the accuracy of the reaching position under various disturbances as a performance index. This idea was based on the fact that older adults show a significant decline in sensory integration in complex motor tasks [10,13,16] and that motor accuracy is widely known to be a valid proxy for measuring sensory integration [13].

In the present study, we investigated whether a decline in sensory integration function in older adults could be detected using a VR-HMD, based on the aforementioned considerations. First, as a preliminary experiment, we confirmed that there were no significant differences in the performance of the designed VR reaching task between young and middle-aged groups. Subsequently, in the primary experiment, we administered the same task to an elderly group and examined whether differences in task performance correlated with rsFC associated with sensory integration function. If rsFC showed correlations aligned with the regions identified in previous studies, the utility of the metrics used in this study is suggested.

## 2. Materials and Methods

### 2.1. Participants

All the participants provided consent to participate in the experiment and allowed the use of their experimental data by submitting a signed consent form. Only individuals without any injuries or conditions that could affect their performance in the VR reaching task were enrolled in the experiment. Although we did not set specific criteria, we ensured that the participants confirmed on the day of the experiment that they had no minor ailments or other issues that could have affected their task performance. In the preliminary experiment, 49 healthy young/middle-aged adults (42 ± 11 years old, 40 males, all right-handed) participated in a VR reaching task. In addition, 40 healthy older adults (77 ± 6 years old, 15 males, all right-handed) participated in the VR reaching task during the main experiment. The inclusion and exclusion criteria were based on the absence of disease at the time of measurement. The participants were instructed to avoid caffeine, alcohol, and strenuous exercise prior to the experiment. The daily physical activity level of the older adult group was determined using a questionnaire and classified as follows: no daily physical activity (level 0); light physical activity, such as walking at least once a week (level 1); strenuous physical activity, such as sports at least once a week (level 2); and strenuous physical activity, such as sports at least three times a week (level 3) (see Tables S1 and S2). This survey was conducted to ensure that the index for the VR reaching task were not influenced by the participants’ recent physical activity levels. The participants were also asked to report any discomfort they felt during the experiment, and all participants reported that the device’s weight did not pose any hindrance or other issues.

The rsfMRI measurements were performed on a group of 40 older adults who performed the VR reaching task, 19 of whom (76 ± 4 years old, 12 males) had no contraindications and agreed to undergo MRI. The time of the visits varied across participants, according to their availability, but there were no correlations between the MRI acquisition time and rsFCs, or between the VR testing time and the mean gap angles indicated in the study. Therefore, this parameter was not included as a covariate in the general linear model analysis used in this study. The time gap between the VR reaching task and the MRI scan varied according to the availability of the participants, ranging from 0 to 60 days (18–20 days). For the participants who performed the tasks on the same day, an MRI scan was first conducted to avoid the effect of the VR reaching task on resting-state brain activity. The research protocol was approved by the Ethical Review Committee for Research Involving Human Subjects of the Tokyo Institute of Technology (approval no. 2021172, 29 November 2021) and was conducted in accordance with the Declaration of Helsinki.

We confirmed that the participants did not have dementia or any other cognitive-function-related diseases and that they could communicate effectively during the experiment and questionnaire survey. Additionally, because we focused on sensory integration related to movement, we did not administer any intelligence tests. However, for the VR reaching task, participants were excluded if their number of correct responses after each trial exceeded three times the standard deviation from the mean or if they made incorrect responses in all trials; they were considered to have misunderstood their duty in the task. However, we also conducted rsFC analysis without applying the exclusion criteria for the VR reaching task; the results are provided in the Supplementary Materials. For fMRI measurements, participants were excluded if visual inspection of distortion-corrected EPI images revealed significant signal loss, indicating insufficient data quality.

### 2.2. VR-HMD Arm-Reaching Task

**Figure 1:**
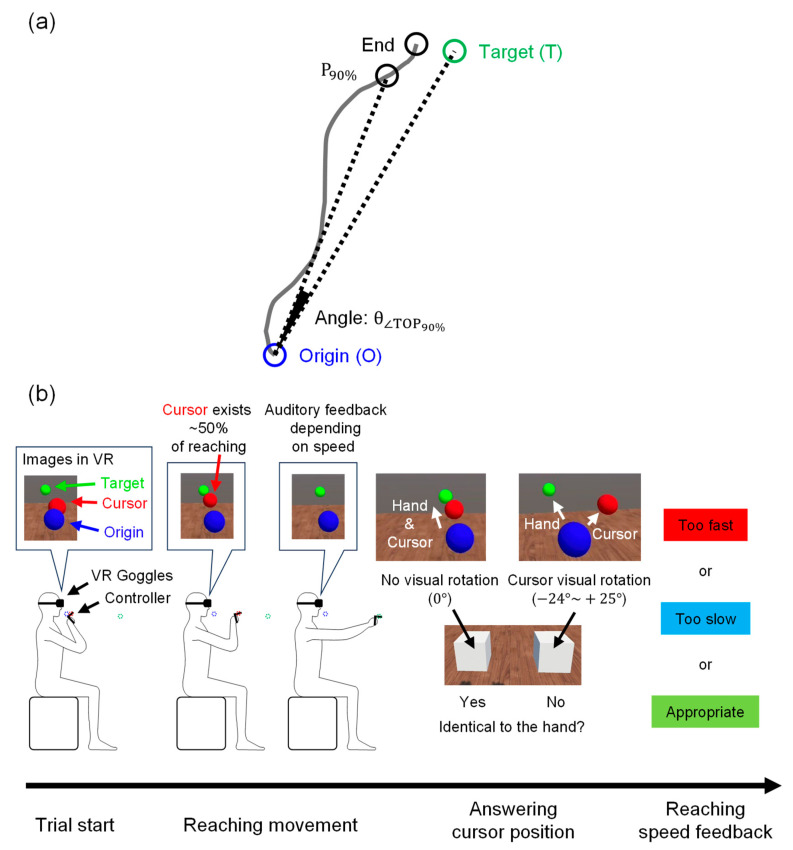
(**a**) Definition of the gap angle: the angle ($\theta_{∠TOP_{90\%}}$) was formed by three points projected on the horizontal plane: 90% position of the reach ($P_{90\%}$), origin (O), and target (T); (**b**) Overview of the VR reaching task: a trial started by positioning the hand controller (red sphere) at the origin (blue sphere) in front of the chin. A target (green sphere) was displayed on an arc centered anteriorly, and the participants reached the target. In some trials, the red sphere was randomly rotated from the actual hand position in the range of −25° to +24°. Further, the red sphere disappeared when the hand was extended by 50% of the distance between the origin and the target. At the end of the reach, sound feedback was provided, according to the reaching speed. To answer whether the rotation was applied during the trial, two white boxes appeared at the end of the trial. Visual feedback on the reaching speed was displayed after the answering time.

Sensory integration function was examined using the gap angle between the hand reaching and target positions (Figure 1a, $\theta_{∠TOP_{90\%}}$) while performing a VR reaching task under multiple cognitive loads. The participants wore a VR HMD (MetaQuest 2, Meta Platforms, Inc., Menlo Park, CA, USA) on their faces and held the two controllers with their hands. In the VR space, they moved a red sphere (i.e., a cursor) representing their right-hand position from a blue sphere (i.e., the origin) to a green sphere (i.e., the target) by moving their right arm (see Figure 1b and Video S1). Because the VR HMD covered the entire field of view, the hands and controllers could not be seen directly. However, the positions of the right hand, origin, and target could be recognized through the VR image. The invisibility of the hands of the VR HMD wearers provided the advantage of being able to perform motor tasks by relying on proprioceptive and visual feedback. The positions of the origin/target were determined based on the arm length through a calibration process performed by each participant prior to initiating the experiment. The origin position was fixed anterior to the chin and the target position was randomly determined per trial on an arc centered anteriorly at 90% of the maximum distance from the origin.

Older adults exhibit a marked decline in sensory integration during complex motor tasks [10,13,16]. To investigate the reaching performance in situations that mimic everyday life, during which participants pay attention to a variety of distractors, we created four specifications that would interfere with reaching. First, the red sphere representing a participant’s right-hand position was visually rotated from its actual position every two or three trials. The rotation angle was randomly selected from 1° to 24° clockwise, or from 1° to 25° counterclockwise, around the vertical upward axis. After each rotated trial, one or two non-rotated trials were performed to wash out the after-effects of the rotation. Second, to prevent reaching by paying attention only to the proprioceptive senses, participants were asked to respond whether the rotation was applied or not after each trial through a question: “Identical to the hand?” Third, the red sphere disappeared when the arm was extended to approximately 50% of its full reach, to prevent answering the question based only on the difference between the end position of reaching and the target position. Fourth, to draw attention towards maintaining a constant reaching speed, auditory feedback was provided at the end of trial depending on the speed deviated from the specified value, and a visual message of “too fast” or “too slow” was displayed.

This experimental application was developed by us using Unity software (version 2021.1.25f1). The APK file was uploaded to the VR-HMD, and the experiment was subsequently conducted. All participants were instructed to reach the target by relying on their own kinesthesia and not on the VR images, and they performed 108 task trials after 10 practice trials to familiarize themselves with the task. The participants were asked to perform a practice session comprising 10 trials. As explained in the basic guidelines of VR technology prior to the practice session, most participants learned to perform the task in a single session. Another session was conducted with participants unable to perform the task correctly after a single practice session. In all trials, hand positions were recorded at a sampling rate of 72 Hz, and answers to questions and target positions were recorded once for each trial.

### 2.3. Behavioral Data Analyses

#### 2.3.1. Mean Gap Angle Calculation

To examine the movement accuracy in the VR reaching task, the mean of the angle ($\theta_{∠TOP_{90\%}}$) was calculated across all trials for each participant (mean gap angle). The reason for using the 90% position rather than the end of the reach was to minimize the effect of a certain number of participants who stopped reaching before reaching the target because of the disappearance of the red sphere in the second half of the reach, which caused an increase in the gap angle and did not reflect their reaching accuracy. In this task, the participants were instructed to extend their arms and reach the target completely. Contrary to expectations, some participants stopped halfway. This could be attributed to the influence of VR on depth perception [17]. However, this task was intended to measure horizontal motor accuracy rather than depth perception accuracy. To maximize the use of the less noisy portion of the data for the desired analysis, we considered 90% of the data to be optimal. The mean gap angle is calculated as follows:

(1) $$Mean\;gap\;angle=\frac{1}{108}\sum_{trial=1}^{108}\left|\theta_{∠TOP_{90\%}}\right|=\frac{1}{108}\sum_{trial=1}^{108}\left|\cos^{-1}\left(\frac{\vec{OP_{90\%}}\cdot \vec{OT}}{\left|\vec{OP_{90\%}}\right|\left|\vec{OT}\right|}\right)\right|,$$

where $\frac{1}{108}\sum_{trial=1}^{108}$ represents taking the average of 108 trials. Additionally, $\vec{OP_{90\%}}$ represents the vector from the origin (O) to 90% of the reach ($P_{90\%}$), and $\vec{OT}$ represents the vector from the origin (O) to the target (T). $\left|\vec{OP_{90\%}}\right|$ and $\left|\vec{OT}\right|$ are the absolute values (vector lengths) of $\vec{OP_{90\%}}$ and $\vec{OT}$, respectively.

We considered the mean gap angle as an index reflecting sensory integration function and used it as an explanatory variable for rsFC analysis using rsfMRI measurement data (see Section 2.5.5).

#### 2.3.2. Statistical Analyses

To analyze the behavioral data, histograms of the mean gap angles were created for the young/middle-aged (preliminary experiment) and older adult groups (main experiment), and a normal distribution test (Shapiro-Wilk test) was conducted. The results confirmed that both groups deviated from normal distribution. Statistical tests were conducted using nonparametric methods. First, for the young/middle-aged group, Spearman’s correlation coefficient was applied to examine whether age and the time of measurement of the VR reaching task were related to the mean gap angle. We also used the Mann–Whitney U test to determine whether there was a significant difference in the mean gap angle between males and females. Next, for the older adult group, we used the same method to check whether age, time of measurement, and physical activity level were related to the mean gap angle and whether there was a significant difference in the mean gap angle between men and women. Furthermore, for the four groups divided by the presence of MRI data and sex, we used the Kruskal-Wallis test to check whether there were significant differences in the mean gap angles between the groups.

### 2.4. MRI Acquisition

A 3 T Magnetom Prisma scanner (Siemens AG, Munich, Germany) was used to acquire the rsfMRI series and structural volumes. The participants were instructed to look at the displayed crosshairs without thinking about specific matters, and two sessions of 6 min resting-state brain activity scans (functional images) and structural images (approximately 5 min) were acquired.

Functional images were further acquired using a T2*-weighted gradient-echo echoplanar imaging sequence (AP and PA phase encode directions) with the following parameters: repetition time (TR) = 800 ms, echo time (TE) = 34.4 ms, flip angle (FA) = 52°, field of view (FOV) = 206.4 × 206.4 mm, matrix size = 86 × 86, 60 slices, slice thickness = 2.4 mm, 450 volumes. Functional images corrected for magnetic distortion were used for the MRI data analysis. For the anatomical MRI acquisition, T1-weighted magnetization-prepared rapid acquisition gradient echo (MP-RAGE) sequence was used with the following parameters (TR = 1.9 ms, TE = 2.52 ms, FA = 9°, FOV = 256 × 256 mm; matrix size = 256 × 256, 224 slices, slice thickness = 1.0 mm).

### 2.5. MRI Data Analyses

We calculated the rsFC using the CONN toolbox [18] (RRID:SCR_009550) release 22.a [19] and SPM [20] (RRID:SCR_007037) release 12.7771.

#### 2.5.1. Preprocessing

Functional and anatomical data were preprocessed using a flexible preprocessing pipeline [21], including realignment with correction of susceptibility distortion interactions, slice-timing correction, outlier detection, direct segmentation, Montreal Neurological Institute (MNI) space normalization, and smoothing. Functional data were realigned using SPM [22] realign and unwarp procedures [23], where all scans were co-registered to a reference image (first scan of the first session) using a least-squares approach and a six-parameter (rigid body) transformation [24], and resampled using b-spline interpolation to correct for motion and magnetic susceptibility interactions. Temporal misalignment between different slices of the functional data (acquired in interleaved order) was corrected following the SPM slice-timing correction (STC) procedure [25,26] using sinc temporal interpolation to resample each BOLD time-series slice to a common mid-acquisition time. Potential outlier scans were identified using artifact detection tools (ART) [27] as acquisitions with framewise displacement above 0.5 mm or global BOLD than three standard deviations [28,29]. A reference BOLD image was computed for each participant by averaging all of the scans and excluding outliers. Functional and anatomical data were normalized to the standard MNI space, segmented into gray matter, white matter, and cerebrospinal fluid (CSF) tissue classes, and resampled to 2 mm isotropic voxels following a direct normalization procedure [28,30] using the SPM unified segmentation and normalization algorithm [31,32] with the default IXI-549 tissue probability map template. Finally, functional data were smoothed using spatial convolution with a Gaussian kernel of 8 mm full width at half maximum (FWHM).

#### 2.5.2. Denoising

The functional data underwent a noise reduction process using a well-established denoising protocol [33]. This protocol involved several steps to mitigate potential confounding effects. These included:

1. Regression of white matter timeseries (utilizing 5 CompCor noise components)
2. Regression of CSF timeseries (also using 5 CompCor noise components)
3. Accounting for motion parameters and their first-order derivatives (12 factors) [34]
4. Removal of outlier scans (up to 41 factors) [29]
5. Adjustment for session and task effects, including their first-order derivatives (two factors)
6. Correction for linear trends (two factors) within each functional run

Following these regression steps, the BOLD timeseries underwent bandpass frequency filtering [35], retaining frequencies between 0.008 Hz and 0.09 Hz. The CompCor [36,37] noise components within the white matter and CSF were derived through a specific process. This involved calculating the average BOLD signal and identifying the principal components orthogonal to this average, the motion parameters, and the outlier scans. These calculations were performed within the eroded segmentation mask of each participant. Given the comprehensive nature of this denoising strategy, the effective degrees of freedom of the BOLD signal after denoising were estimated. These estimates ranged from 103.4 to 110.1 across all participants, with an average of 108.4 [28]. This thorough denoising approach aimed to enhance the signal quality and reduce potential confounds in the functional data, thereby improving the reliability of the subsequent analyses.

#### 2.5.3. Regions of Interest (ROIs)

For this investigation, we used the atlas provided by CONN [18] (RRID:SCR_009550) version 22.a [19]. To enhance the precision of our analysis in motor and sensory regions, we substituted the precentral and postcentral gyri with 12 sensorimotor area templates (six for each hemisphere), as defined in the Human Motor Area Template (HMAT [38]). This modification resulted in 140 ROIs. Previous research has demonstrated that motor control in elderly individuals often involves neural activity that extends beyond the brain areas traditionally linked to motor function [39]. This expanded activation pattern is thought to be a consequence of two age-related phenomena: brain dedifferentiation [40] and compensatory mechanisms [41]. Given these considerations, our study utilized a comprehensive whole-brain atlas. This method allowed us to explore a broad range of brain regions, enabling us to identify areas that contributed to the observed decline in sensory integration in old age. By adopting this whole-brain perspective, we aimed to capture both conventional and potentially novel brain areas involved in sensorimotor processing in older adults. This approach acknowledges the complex and potentially widespread neural changes that occur with aging, particularly in the context of sensorimotor functions.

#### 2.5.4. First-Level Analysis

To analyze patterns of functional connectivity across the 140 ROIs, we employed two methods: seed-based connectivity maps (SBC) and ROI-to-ROI connectivity matrices (RRCs). The strength of functional connectivity was quantified using Fisher-transformed bivariate correlation coefficients. These coefficients were derived from a weighted general linear model (weighted GLM [42]), which was computed separately for each pair of seed and target areas. This model was designed to assess the relationship between the BOLD signal time series of paired regions. To account for potential transient magnetization effects that may occur at the onset of each run, we implemented a weighting strategy for the individual scans. This involved the use of a step function, which was convolved with an SPM canonical hemodynamic response function and was subsequently rectified. This approach allowed us to characterize functional connectivity patterns across the brain while mitigating potential confounding factors related to scan acquisition. Using both the SBC and RRC methods, we were able to capture a comprehensive picture of the functional relationships between different brain regions. The weighted-GLM approach provides a robust framework for quantifying these relationships, considering the temporal dynamics of the BOLD signal. This method enabled us to generate detailed connectivity maps and matrices, offering insight into the functional architecture of the brain across the studied ROIs.

#### 2.5.5. Group-Level Analyses

Group-level analyses were performed using general linear models (GLM). In these analyses, the RRCs obtained from the first-level analysis served as dependent variables, whereas age, sex, physical activity level, and mean gap angle calculated from the behavioral data analysis were used as independent variables. This approach aimed to estimate the functional connections between different ROIs that were highly correlated with the mean gap angle. The results were tested using an FDR-corrected *p*-value of <0.05. This methodology allowed us to identify functional connections that significantly correlated with the mean gap angle for C2140  pairs, while controlling for the confounding effects of age, sex, and physical activity level.

## 3. Results

### 3.1. Behavioral Difference between Older Adults and Young/Middle-Aged Adults

None of the participants in either the preliminary or main experiments were ill at the time of measurement. The daily physical activity levels for older adults were distributed as follows: six at level 0, 24 at level 1, seven at level 2, and three at level 3. Eight participants in the young/middle-aged group (preliminary experiment) were excluded from the analysis based on the exclusion criteria. In the older adult group (main experiment), five participants were excluded from the analysis. Finally, 41 participants (43 ± 11 years old, 33 males) from the young/middle-aged group and 35 participants (76 ± 5 years old, 12 males) from the older adult group were analyzed. Detailed information is summarized in Tables S1 and S2.

**Figure 2:**
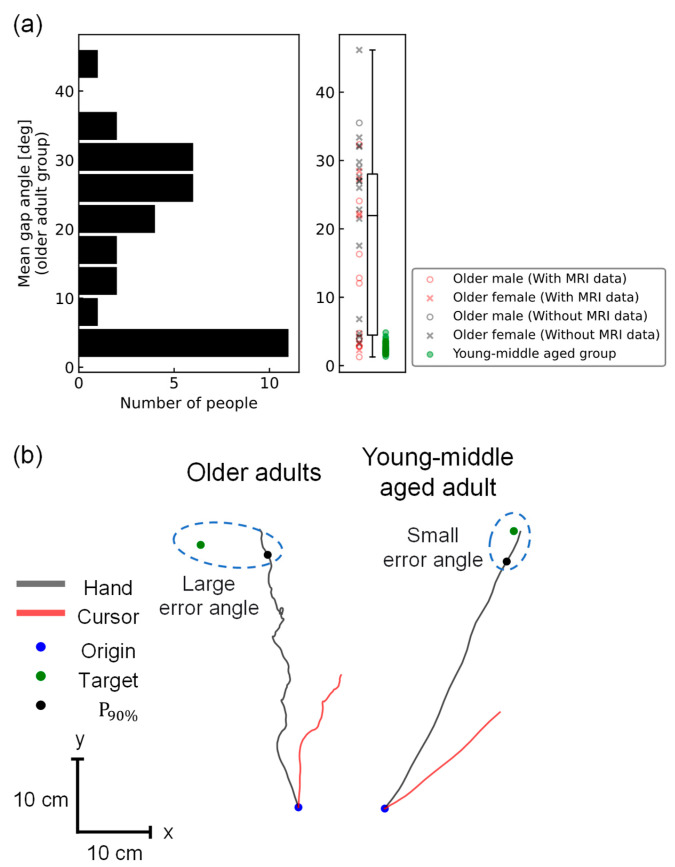
(**a**) Histograms, scatter plots, and box plots of the mean gap angles in the older adult group (this experiment), with males represented by 〇 and females by ×. Participants with MRI data are shown in red, and those without are shown in black. There were no significant differences between men and women, or among the four groups (between men and women: Mann–Whitney U-test, U = 85, *p* = 0.07; among the four groups: Kruskal-Wallis test, H = 5.8, *p* = 0.12). Scatter plots for the young/middle-aged group (preliminary experiment) are shown in green to illustrate the large individual differences in the older adult group; (**b**) Representative reaching trajectories of a single trial in the older adult and young/middle-aged groups. The reaching trajectories are shown as black lines, and the cursor trajectories as red lines (when the visuomotor rotation angle is −22°). Participants in the older adult group tended to reach by meandering away from the target, whereas those in the young/middle-aged group reached straight toward the target.

Movement accuracy, defined as the mean gap angle between the reached hand and the target positions, was calculated for each participant. For the young/middle-aged group (preliminary experiment), the mean ± standard deviation of the mean gap angle was 2.5 ± 0.8° (Figure 2a). There was no significant correlation between the mean gap angle and either age or time of measurement in the VR reaching task (age: Spearman’s r = 0.01, *p* = 0.9; time: Spearman’s r = 0.07, *p* = 0.7). There was no significant difference in the mean gap angle between males and females (U-statistic = 149, *p* = 0.6). For the older adult group (this experiment), the mean ± standard deviation of the mean gap angle was 18.5 ± 12.6° (Figure 2a). There was no significant correlation between the mean gap angle and age, time of measurement, or physical activity level (age: Spearman’s r = 0.08, *p* = 0.6; time: Spearman’s r = 0.04, *p* = 0.8; physical activity level: Spearman’s r = −0.14, *p* = 0.4). There was also no significant difference in the mean gap angle between men and women (U-statistic = 85°, *p* = 0.07). Furthermore, there was no significant difference in the mean gap angle among the four groups divided by sex and availability of MRI data (statistic = 5.8, *p* = 0.12). While all the participants in the young/middle-aged group showed angles of less than 5 °, the measurements in the older adult group varied widely, with 12 participants having <10° and 21 having >20°. Participants with a large mean gap angle were unable to reach the target straight and tended to reach it by meandering away from it (Figure 2b). The individual mean map angles are listed in Table S3.

### 3.2. rsFC of Older Adults Reflecting the Mean Gap Angle

Of the nineteen older adult participants in the MRI experiment, three were excluded from the analysis based on the exclusion criteria. Consequently, rsFC was analyzed for 16 participants (average age 76 ± 4 years, 10 males) in the older adult group. Detailed information is provided in Tables S1 and S2.

**Figure 3:**
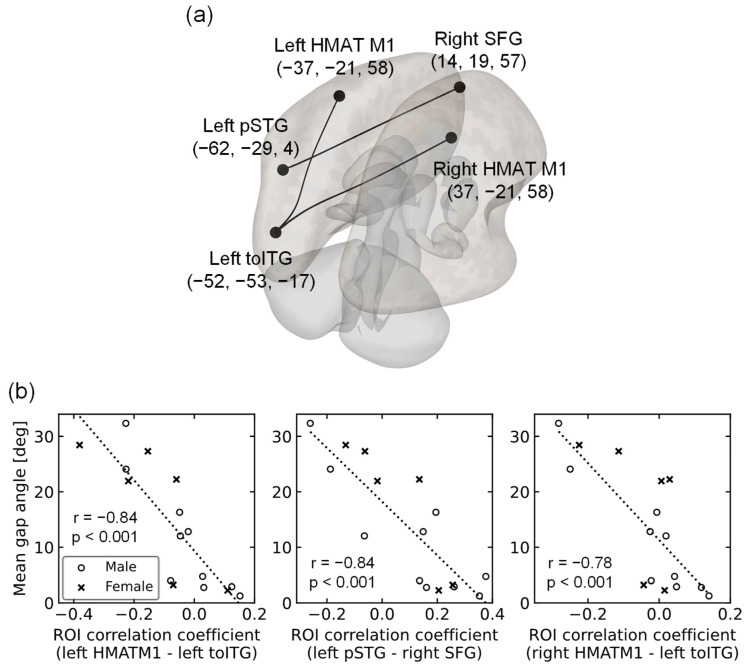
(**a**) Three pairs of rsFCs showing significant correlations with the corresponding mean gap angles. One is the connection between the left HMAT M1 and the left inferior temporal gyrus, temporo-occipital part (toITG), and the second is the connection between the left superior temporal gyrus, posterior division (pSTG), and right superior frontal gyrus (SFG). The third is the connection between the right HMAT M1 and the left inferior temporal gyrus, temporo-occipital part (toITG). Numbers represent the position of the center of gravity in the MNI coordinate system; (**b**) Scatterplot of the mean gap angle and ROI correlation coefficient for 16 participants. 〇 indicate males, and × indicates females. The top figure shows the connection between left HMAT M1 and left toITG, the middle figure shows the connection between left pSTG and right SFG, and the bottom figure shows the connection between right HMAT M1 and left toITG. The dotted lines represent approximate straight lines.

The three pairs of rsFCs significantly correlated with the mean gap angle. The first indicated the association of the left HMAT M1 with the left inferior temporal gyrus, temporooccipital part (toITG) (T(12) = −5.60, p-FDR = 0.02); the second was the association of the left superior temporal gyrus, posterior division (pSTG), and right superior frontal gyrus (SFG) binding (T(12) = −5.69, p-FDR = 0.02); and the third was the right HMAT M1 and binding of the left inferior temporal gyrus and temporooccipital part (toITG) (T(12) = −4.98, p-FDR = 0.03) (see Figure 3). All were negatively correlated, showing a greater functional connectivity strength in participants with higher motor accuracy, that is smaller mean gap angles.

## 4. Discussion

In this study, to propose a task capable of assessing sensory integration in daily life at home, we examined whether VR-HMD-based reaching task performance was associated with rsFC between the brain regions involved in sensory integration. The task performance was further assessed using the gap angle between the hand-reaching and target positions (i.e., the mean gap angle) during the VR reaching task with multiple cognitive loads. As expected, the young/middle-aged group showed small errors and small individual differences, less than 5° for all participants, whereas the older adult group showed extensive individual differences, with 12 participants having angles of less than 10° and 21 participants having angles of more than 20°. Participants in the older adult group, who had lower motor accuracy, tended to reach by meandering away from the target, whereas those in the young/middle-aged group reached straight towards the target (Figure 2b). Furthermore, in the older adult group, a significant correlation was observed between the gap angle and rsFC between the left/right HMAT M1 and the left ITG, indicating that the integrated function of motor control and visual information processing is involved in the decline in sensory integration in old age. In particular, as the rsFC between the left HMAT M1 and the left ITG survived even without including daily physical activity level as a covariate of GLM (T(12) = −5.55, p-FDR = 0.02), this rsFC would be the best index to detect a decline in sensory integration function in old age. Based on these results, daily arm-reaching movements can be used as an index of declining sensory integration in old age. For routine detection, it is necessary to construct a system using an off-the-shelf, economical device that can be used at home rather than KINARM, which is a large, research-grade, and extremely expensive device. This study demonstrates that it is possible to construct a system that satisfies these requirements, detecting functional decline without requiring a large space for experiments, expensive equipment, or specialized knowledge. Although studies have utilized VR systems in the rehabilitation of patients with stroke and spinal cord injury [14,15], no studies have yet examined the use of VR systems to assess individual differences in reductions in sensory integration among healthy elderly individuals. Therefore, we believe that the system used in this study will be particularly useful.

In the present study, the mean ± standard deviation of the gap angle for the young/middle-aged group was 2.5 ± 0.8°, whereas the older adult group showed a larger variation of 18.5 ± 12.6°. There was no significant correlation between the mean gap angle and age in either group (young/middle-aged group: Spearman’s r = 0.01, *p* = 0.9; older adult group: Spearman’s r = 0.08, *p* = 0.6). These findings suggest that the mean gap angle detects individual differences in motor function in the elderly owing to a decline in sensory integration function in old age. Additionally, there was no significant correlation between the variation in the older adult group and the physical activity level (Spearman’s r = −0.14, *p* = 0.4). This indicates that the mean gap angle is independent of recent physical activity levels and reflects the sensory integration function of the participants. Although various indices that indicate differences between young/middle-aged, and older adult groups have been studied [5,6,7,9,11,13], to the best of our knowledge, no studies have identified indices for individual differences in the decline of sensory integration in healthy older adults, and we consider the mean gap angle to be very useful.

It should be noted that the participants included in the analysis of this study comprised 33 males and 8 females (80% male proportion) in the young/middle-aged group and 12 males and 23 females (34%) in the older adult group, representing a clear difference in the sex ratio for each group. Regarding sex differences in fine motor movements, studies have shown differences in motor tendencies during computer-pointing tasks [43]. In contrast, a study examining sex differences in gross and fine motor movements and the effects of aging found no significant sex differences in any of these movements, indicating a strong effect of age [44]. In this study, there was no significant difference in the mean gap angle between men and women in both the young/middle-aged and older adult groups (young/middle-aged group: U-statistic = 149, *p* = 0.6; older adult group: U-statistic = 85, *p* = 0.07). Based on these results, we believe that the mean gap angle is useful, even when sex bias is considered.

Next, assuming that the decline in sensory integration in old age is related to resting-state brain activity, three rsFCs that significantly correlated with motor accuracy were identified in older adults. The first was the connection between the left HMAT M1 and left ITG. The Left HMAT M1 is the left primary motor cortex [38] and is thought to play a major role in motor control of the right arm [45]. The left ITG is thought to play an important role in visual information processing. For example, the hemodynamics of the left ITG change during active work and passive observation in a visual information task, suggesting that the left ITG may be involved in visual information processing [46]. The functional connectivity between these two regions tended to be stronger in older adults with higher motor accuracy, indicating that the integration of motor control and visual information processing through this connection is related to a decline in sensory integration function in older adults. Visual processes such as visual acuity and depth perception have been found to decline with age [3]. Age-related declines in motor skills that require vision, such as catching a ball [10] and reaction time to visual stimuli [16], have also been reported. Therefore, we considered this connection to increase the reliability of mean gap angle as a measure of sensory integration. In addition, this connection was obtained from individual differences in the visuomotor abilities of healthy older adults, unlike the comparison between young/middle-aged, and older adult groups in previous studies. The mean gap angle may serve as a new index for individual differences in the decline of sensory integration in healthy older adults [3,10,16].

The second important connectivity was the connection between the left pSTG and right SFG. The left pSTG plays an important role in phonological processing [47,48] and the right SFG controls motor inhibition [49]. The functional connectivity between these two areas also tended to be stronger in older adults with higher motor accuracy, suggesting that the connection between phonological processing and motor inhibitory control is related to a decline in sensory integration in older adults. However, previous studies have not yet implicated phonological processing in the decline of movement-related sensory integration. Therefore, we considered two interpretations of this connection. The first was the effect of age-related brain dedifferentiation and compensation. Dedifferentiation is defined as the loss of functional brain localization and the diffusion of activity throughout the brain due to age-related changes in neurotransmission [40]. Compensation refers to compensating for the loss of function by activating more brain regions as we age [41]. These phenomena raise the possibility that activities other than those in brain regions generally thought to be involved in motor control may also be involved in sensory integration during old age. Several studies have suggested the dedifferentiation and compensation of the motor system [39,50,51]. This dedifferentiation and compensation may improve motor control by increasing left and right pSTG activity and compensating for the reduced motor system function. The second factor was the effect of task dependence. In the present study, the procedures for each step of the motor task were explained using visual messages in text in the VR space and auditory messages in the audio. Further research is required to determine whether this connection is derived from understanding the task explanation in a motor task without using these messages.

The third important connectivity was between the right HMAT M1 and left ITG. The right HMAT M1 is the right primary motor cortex [38] and is thought to play a major role in motor control, particularly in the left arm. However, it has also been found to be involved in the motor control of the right arm, and it has been noted that the involvement of the ipsilateral arm of the primary motor cortex in control increases with age [52,53]. One study performing fMRI measurements during a tapping task with the fingers of the right hand in various age groups confirmed that the amount of bilateral M1 activation increased with age, with a significant increase in ipsilateral M1 activation [52]. In addition, a study using transcranial magnetic stimulation (TMS) showed that stimulation of the ipsilateral primary motor cortex had no effect on the younger group, but increased the reaction time in the older group [53]. These studies suggested that primary motor cortex dedifferentiation and compensation intensify with age. This third connectivity tends to be stronger in older adults with higher motor accuracy, suggesting that it functions in the dedifferentiation and compensation of the right primary motor cortex. The integration of motor control and visual information processing plays an important role in enhancing sensory integration during old age. We believe that this connectivity supports the idea that mean gap angle is a useful index of declining sensory integration in older adults.

In the present study, a VR reaching task was proposed as a system for detecting the age-related decline in sensory integration. It has been suggested that the mean gap angle during a task can serve as an effective index of functional decline. For the practical application of this index to detect sensory integration function in old age, it is necessary not only to examine hand-reaching movements using the VR reaching task in this study but also to verify the relevance of the index to the decline in motor functions, such as walking and stair climbing, that older adults experience in daily life. Future research should investigate the extent of the relationship between mean gap angle and motor control abilities in real space. In addition, because the task was designed with a focus on motor accuracy, only the mean gap angle was used as an index to evaluate movement, to avoid complicating the discussion. However, in VR-based exercise tasks, other factors (e.g., reaction speed and growth rate) may also be used as indices of decline in sensory integration function in old age. These issues should further be investigated in relation to real-space movements. There is also room for further examination of task difficulty levels. The present study confirmed that individual differences were small in the young/middle-aged group, but large in the older adult group. However, determining whether a task’s difficulty level is optimal for detecting a decline in sensory integration in old age requires further investigation, such as comparing tasks with varying difficulty levels. Because the physical activity levels in this study were based on the participants’ self-reports, they may have been biased by factors such as measurement errors [54], social desirability, or social approval [55]. In addition, the time between the VR reaching task and MRI measurements in this study ranged from 0 to 60 days, depending on the participant. Given that older adults undergo significant brain changes within a short period, even in healthy subjects [56], differences in the measurement period for each participant may have affected the results. It should also be noted that the rsFC analysis in the present study was performed only in older adults. Although rsFC analysis detected significant connections related to the variation in the mean gap angle in the elderly, whether this connection supports the decline in sensory integration function in old age remains to be verified. The observed significance of the left ITG, while the cerebellar cortex and angular gyrus, which are often reported in visuomotor tasks, were not significant, is intriguing and requires further investigation. Zwergal et al. suggested that aging maintains network function in the cerebellum and other regions, but alters network function in the sensory system [57], which is expected to have an impact. However, further analysis involving the young/middle-aged group is required to verify these findings.

In conclusion, this study proposed a VR reaching task system designed to detect declines in sensory integration function among older adults. Index derived from the VR reaching task revealed individual differences in motor accuracy among older adults, showing significant correlations with the rsFC related to sensory integration. Specifically, a significant correlation was observed between the connectivity of the left and right HMAT M1 and that of the left to the ITG. Although the rsFC analysis in this study focused solely on the older adult group, further validation with a larger sample size, including a young/middle-aged group, is required. Nonetheless, our findings suggest that the proposed VR system has the potential to detect age-related decline in motor control abilities in daily life.

## Data Availability

The data supporting the findings of this study are available from the corresponding author (N.Y.) upon reasonable request. The data are not publicly available due to ethical restrictions.

## Supplementary Materials

The following supporting information can be downloaded at: https://www.mdpi.com/article/10.3390/brainsci14080840/s1, Table S1: Participant information for the young/middle-aged group. * indicates that the participant was excluded from the analysis because the number of false responses in trials with or without visuomotor rotation was more than three standard deviations above the mean of the responses after each trial of the VR reaching task. ** indicates that the participant was excluded from the analysis because they answered incorrectly on all trials with or without visuomotor rotation; Table S2: Participant information for the older adult group. *** indicates that the participant was excluded from the analysis of the fMRI measurements because the EPI images showed an obvious area of signal loss in the orbitofrontal region upon visual inspection; Table S3: Mean gap angle of the VR reaching task for individual participants; Figure S1: Color map illustrating the strength of connections between the left HMAT M1 and other brain regions. In the color bar, red and orange indicate positive connections, whereas blue and cyan indicate negative connections; Figure S2: Color map illustrating the strength of connections between the right HMAT M1 and other brain regions. In the color bar, red and orange indicate positive connections, whereas blue and cyan indicate negative connections; Figure S3: Results of the rsFC analysis conducted on data from 18 participants, without applying any exclusion criteria for the VR reaching task. (**a**) Two rsFC pairs showed significant correlations with the mean gap angle: first, the connection between the left HMAT and M1 and the left toITG (T(13) = −5.62, p-FDR = 0.01); second, the connection between the right HMAT and M1 and the left toITG (T(13) = −4.80, p-FDR = 0.05). The numbers indicate the positions of the centers of gravity in the MNI coordinate system. (**b**) Comparison between the rsFC analysis with 16 participants and that with 18 participants. Similar to the analysis with 16 participants, the analysis with 18 participants also revealed significant left-to-right HMAT-M1 and left-to-ITG connections; Video S1: VR-HMD recording during the VR reaching task. The participants moved their right hand from the blue sphere to the green sphere. After reaching, they predicted the presence of a visuomotor rotation in each trial.
