# Supplementary material for: Decline in Sensory Integration in Old Age and Its Related Functional Brain Connectivity Correlates Observed during a Virtual Reality Task
Source: Brain Sci. 2024 Aug 21;14(8):840. doi: 10.3390/brainsci14080840 (PMC11352474; doi:10.3390/brainsci14080840)
Supplement: Supplementary file 1 [file brainsci-14-00840-s001.zip › brainsci_Inagaki_SupplementaryMaterials.pdf]

brain sciences

MDPI

Table S1. Participant information for the young-middle-aged group. * indicates that the participant was excluded from the analysis because the number of false responses in the trials with or without visuomotor rotation was more than three standard deviations above the mean in the responses after each trial of the VR-HMD reaching task. ** indicates that the participant was excluded from the analysis because they answered incorrectly on all trials with or without visuomotor rotation.

No.	Age	Gender	Number of incorrect answers (with Rotation trial)	Number of incorrect answers (without Rotation trial)	Exclusion (Reaching task)
YM1	56	F	12/49	5/59	
YM2	49	M	9/49	2/59	
YM3	25	M	12/49	18/59	
YM4	45	M	20/49	0/59	
YM5	46	M	24/49	2/59	
YM6	50	M	13/49	0/59	
YM7	39	M	49/49	0/59	Yes**
YM8	42	F	21/49	2/59	
YM9	30	F	25/49	5/59	
YM10	30	M	24/49	5/59	
YM11	56	M	19/49	5/59	
YM12	43	M	0/49	59/59	Yes**
YM13	35	M	43/49	1/59	
YM14	53	M	46/49	1/59	
YM15	56	M	30/49	0/59	
YM16	54	M	17/49	5/59	
YM17	51	M	49/49	0/59	Yes**
YM18	26	M	30/49	0/59	
YM19	35	M	21/49	5/59	
YM20	59	M	16/49	6/59	
YM21	48	M	40/49	0/59	
YM22	46	F	19/49	3/59	
YM23	27	M	23/49	0/59	
YM24	41	M	38/49	1/59	
YM25	39	M	24/49	3/59	
YM26	26	M	37/49	10/59	
YM27	56	F	47/49	3/59	
YM28	54	M	29/49	1/59	
YM29	33	F	40/49	4/59	
YM30	46	M	26/49	3/59	
YM31	33	M	11/49	0/59	
YM32	38	M	17/49	14/59	
YM33	32	F	47/49	2/59	

YM34	53	M	23/49	3/59	
YM35	51	F	19/49	43/59	Yes*
YM36	49	M	18/49	2/59	
YM37	55	M	48/49	0/59	
YM38	54	F	18/49	1/59	
YM39	55	M	18/49	1/59	
YM40	41	M	16/49	0/59	
YM41	41	M	41/49	0/59	
YM42	39	M	49/49	1/59	Yes**
YM43	27	M	49/49	0/59	Yes**
YM44	26	M	49/49	0/59	Yes**
YM45	55	M	49/49	0/59	Yes**
YM46	36	M	17/49	1/59	
YM47	47	M	24/49	1/59	
YM48	26	M	22/49	0/59	
YM49	23	M	22/49	11/59	

Table S2. Participant information for the the older adult group. *** indicates that the participant was excluded from the analysis of the fMRI measurements because the EPI images showed an obvious area of signal loss in the orbitofrontal region upon visual inspection.

No.	Age	Gender	Daily physical activity level	Number of incorrect answers (with Rotation trial)	Number of incorrect answers (without Rotation trial)	Exclusion (Reaching task)	MRI	Exclusion (fMRI)
O1	73	F	3	15/49	2/59		○	
O2	70	F	3	38/49	1/59		○	
O3	74	M	1	13/49	8/59		○	
O4	76	M	1	28/49	1/59		○	
O5	75	M	0	49/49	0/59	Yes**	○	
O6	79	M	1	20/49	14/59		○	
O7	97	F	0	7/49	53/59	Yes*	×	
O8	73	F	1	20/49	9/59		×	
O9	81	F	0	44/49	4/59		×	
O10	79	F	1	18/49	8/59		○	
O11	79	M	1	16/49	2/59		○	
O12	85	M	2	25/49	20/59		×	
O13	82	F	1	24/49	14/59		×	
O14	82	F	2	23/49	29/59		×	
O15	75	F	1	17/49	14/59		×	
O16	81	F	0	24/49	16/59		×	
O17	83	F	1	28/49	6/59		○	Yes***
O18	74	F	2	22/49	3/59		×	

O19	78	F	1	23/49	11/59		○
O20	71	F	1	22/49	2/59		×
O21	67	F	2	33/49	2/59		×
O22	69	F	2	20/49	16/59		×
O23	74	F	1	19/49	1/59		×
O24	73	F	1	32/49	13/59		×
O25	72	F	1	21/49	7/59		×
O26	74	M	1	15/49	3/59		×
O27	84	F	1	34/49	4/59		×
O28	71	F	1	18/49	6/59		×
O29	77	M	1	49/49	0/59	Yes**	○
O30	72	M	0	49/49	1/59	Yes**	×
O31	85	F	1	17/49	7/59		×
O32	84	F	1	45/49	6/59		×
O33	72	M	3	12/49	0/59		○
O34	83	M	0	43/49	1/59		○
O35	77	F	1	15/49	9/59		○
O36	71	M	2	12/49	32/59		○
O37	71	M	1	16/49	2/59		○
O38	74	F	1	34/49	2/59		○
O39	74	M	1	13/49	23/59		○
O40	82	M	2	9/49	5/59		○

Table S3. Mean gap angle of the VR task for individual participants.

No.	Mean gap angle	No.	Mean gap angle	No.	Mean gap angle	No.	Mean gap angle
O1	3.2	O22	6.8	YM1	2.4	YM25	2.1
O2	28.4	O23	3.2	YM2	2.1	YM26	2.5
O3	4	O24	32.2	YM3	2.1	YM27	2.2
O4	2.8	O25	29.8	YM4	2.4	YM28	3.2
O6	12	O26	3.9	YM5	3.6	YM29	3.1
O8	4.7	O27	27	YM6	1.7	YM30	2.8
O9	46.2	O28	27.6	YM8	2.2	YM31	1.3
O10	2.3	O31	22.8	YM9	4.8	YM32	2.5
O11	1.3	O32	26	YM10	3.5	YM33	1.9
O12	35.5	O33	2.9	YM11	2	YM34	2.4
O13	28.9	O34	32.4	YM13	2.7	YM36	1.9
O14	4.2	O35	22.2	YM14	4.2	YM37	2.7
O15	17.5	O36	12.8	YM15	3.2	YM38	1.9
O16	21.5	O37	16.3	YM16	2.4	YM39	2.4
O18	27	O38	21.9	YM18	3.3	YM40	2.1

O19	27.3	O39	4.7	YM19	2.6	YM41	1.7
O20	32	O40	24.1	YM20	3.5	YM46	2.2
O21	33.4			YM21	2.9	YM47	1.8
				YM22	1.7	YM48	1.6
				YM23	1.8	YM49	3.9
				YM24	2.4		

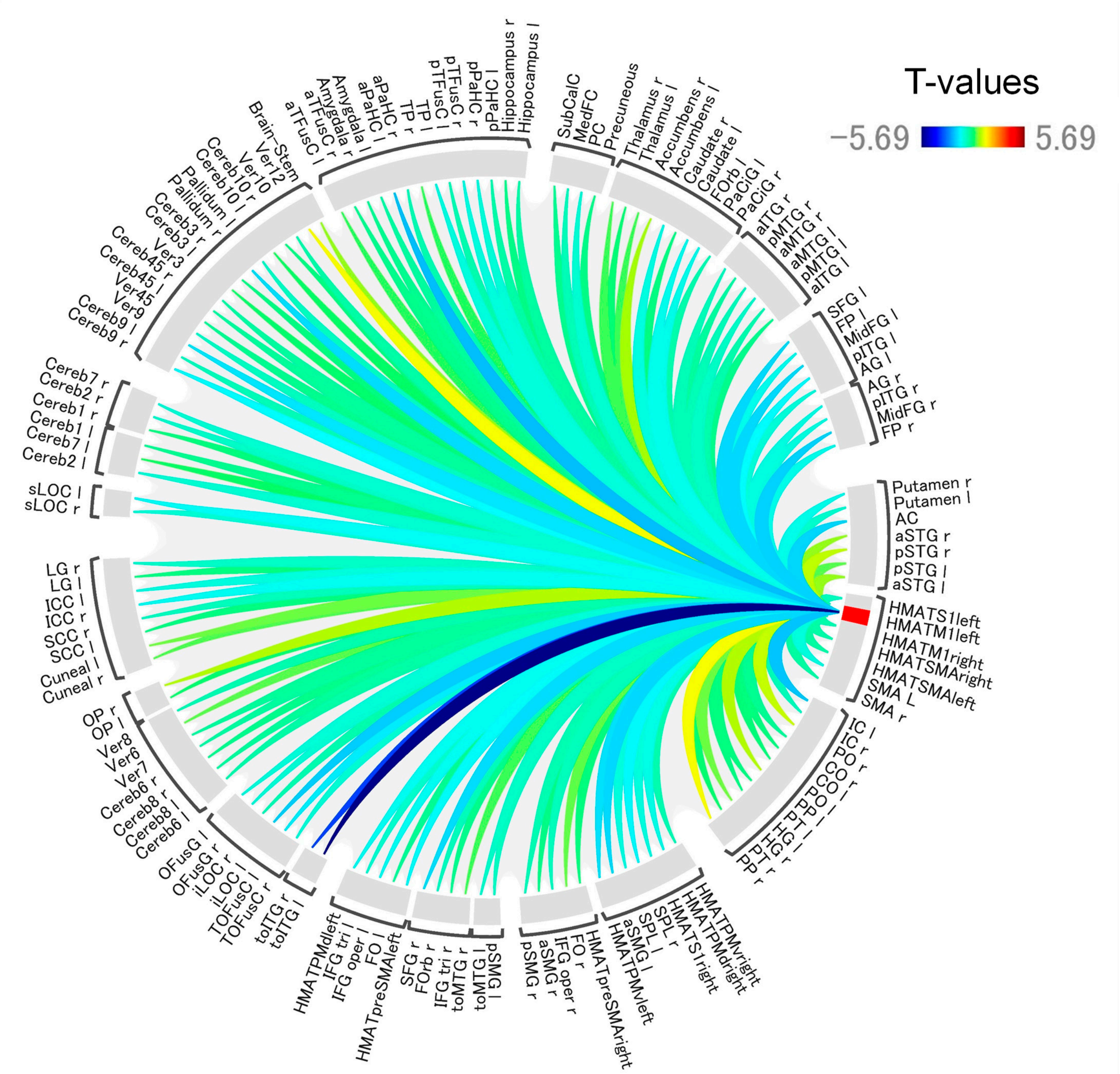

Figure S1: Color map illustrating the strength of connections between the left HMAT M1 and other brain regions. In the color bar, red and orange indicate positive connections, whereas blue and cyan indicate negative connections.

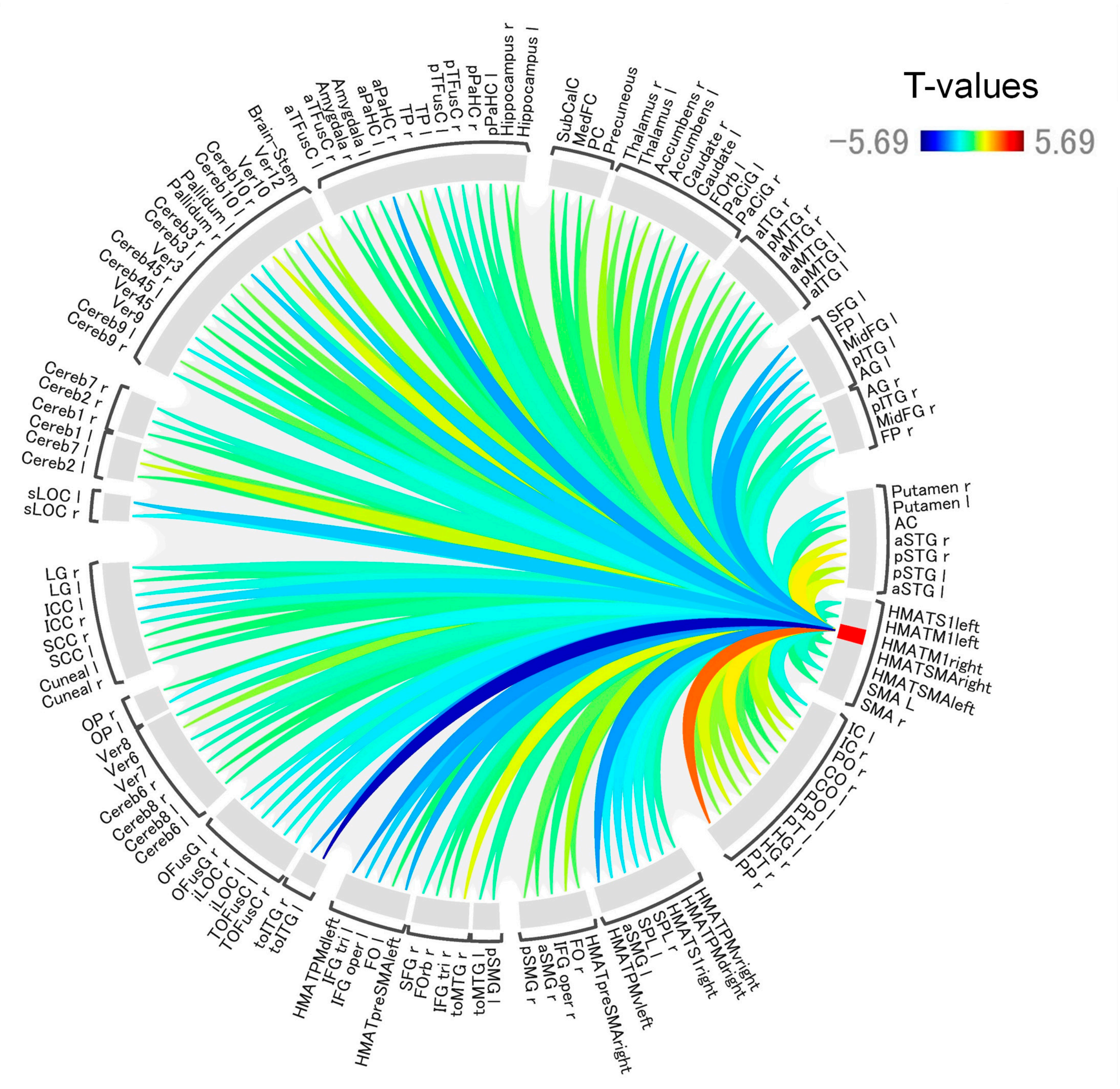

Figure S2: Color map illustrating the strength of connections between the right HMAT M1 and other brain regions. In the color bar, red and orange indicate positive connections, whereas blue and cyan indicate negative connections.

(a)

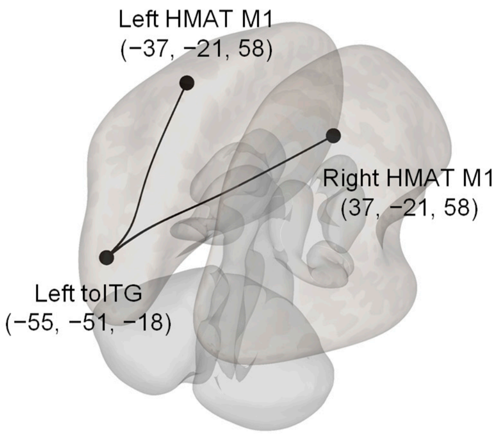

(b)

	significant connection	t-value	p-FDR
Analysis with 16 participants	left pSTG - right SFG	T(12) = −5.69	0.02
	left HMAT M1 - left toITG	T(12)= −5.60	0.02
	right HMAT M1 - left toITG	T(12) = −4.98	0.03
Analysis with 18 participants	left HMAT M1 - left toITG	T(13) = −5.62	0.01
	right HMAT M1 - left toITG	T(13) = −4.80	0.05

Figure S3: Results of the rsFC analysis conducted on data from 18 participants, without applying any exclusion criteria for the VR reaching task. (a) Two rsFC pairs showed significant correlations with the mean gap angle: first, the connection between the left HMAT and M1 and the left toITG (T(13) = −5.62, p-FDR = 0.01); second, the connection between the right HMAT and M1 and the left toITG (T(13) = −4.80, p-FDR = 0.05). The numbers indicate the positions of the centers of gravity in the MNI coordinate system. (b) Comparison between rsFC analysis with 16 participants and that with 18 participants. Similar to the analysis with 16 participants, the analysis with 18 participants also revealed significant left-to-right HMAT-M1 and left-to-ITG connections.

Video S1: VR-HMD recording during the VR reaching task. The participants moved their right hand from the blue sphere to the green sphere. After reaching, they predicted the presence of a visuomotor rotation in each trial.

List of Abbreviations:

- Virtual Reality Head-Mounted Display (VR-HMD)
- Resting-State Functional Connectivity (rsFC)
- Resting-State Functional Magnetic Resonance Imaging (rsfMRI)
- Montreal Neurological Institute (MNI)
- Cerebrospinal Fluid (CSF)
- Regions of Interest (ROIs)
- Human Motor Area Template (HMAT)
- ROI-to-ROI Connectivity Matrices (RRCs)
- General Linear Model (GLM)
